# The effect of vitamin C in adults with sepsis: a meta-analysis of randomized controlled trials

**DOI:** 10.3389/fmed.2023.1244484

**Published:** 2023-08-31

**Authors:** Xing Luo, Youfeng Zhu, Rui Zhang, JianQiu Zhu, Huanming Kuang, Yuebin Shao, Xinmin Guo, Bo Ning

**Affiliations:** ^1^Department of Intensive Care Unit, Guangzhou Red Cross Hospital, Jinan University, Guangzhou, China; ^2^Department of Traditional Chinese Medicine, Guangzhou Red Cross Hospital, Jinan University, Guangzhou, China; ^3^Department of Ultrasonography, Guangzhou Red Cross Hospital, Jinan University, Guangzhou, China; ^4^Department of Neurosurgery, Guangzhou Red Cross Hospital, Jinan University, Guangzhou, China

**Keywords:** sepsis, vitamin C, mortality, meta-analysis, treatment

## Abstract

**Background:**

The effect of intravenous (IV) vitamin C in the treatment of sepsis remains controversial. We aimed to explore the clinical efficacy of vitamin C in the treatment of sepsis.

**Methods:**

Electronic databases (PubMed, Embase, Web of Science, and Cochrane Library) were searched from inception through November 15th, 2022, for randomized controlled trials evaluating the effect of IV vitamin C treatment in patients with sepsis. The primary outcome was short-term mortality, secondary outcomes included duration of vasopressor use, length of intensive care unit (ICU) stay, and Sequential Organ Failure Assessment (SOFA) score after vitamin C treatment. Subgroup analyses were performed based on the dose and duration of IV vitamin C and region to determine whether vitamin C benefited patients with sepsis.

**Results:**

A total of 10 studies including 1,426 patients fulfilled the predefined criteria and were analyzed. Overall, there were no significant differences between the vitamin C group and the control group regarding short-term mortality [odds ratio (OR), 0.61; 95% confidence interval (CI) 0.37–1.01; *p* = 0.05], ICU length of stay [mean difference (MD), −1.24; 95% CI -3.54 to 1.05, *p* = 0.29] and SOFA score (MD, −0.85, 95% CI -2.38 to 0.67, *p* = 0.27). However, vitamin C significantly reduced the duration of vasopressor use (MD, −14.36, 95% CI −26.11 to −2.61, *p* = 0.02). Furthermore, subgroup analysis found that in developing countries, vitamin C was associated with a significant reduction in short-term mortality (OR, 0.33; 95% CI 0.12–0.90; *p* = 0.03), duration of vasopressor use (MD, −24.37, 95% CI -33.72 to −15.02, *p* < 0.001) and SOFA score (MD, −2.55, 95% CI -4.81 to −0.28, *p* = 0.03).

**Conclusion:**

In our study, vitamin C administration for sepsis patients was not associated with a significant reduction in short-term mortality, length of ICU stay or SOFA score. However, we observed that vitamin C could reduce the duration of vasopressor use. Furthermore, sepsis patients in developing countries may benefit more from vitamin C administration than those in developed countries.

**Systematic review registration:** Identifier CRD42022380958, https://www.crd.york.ac.uk/PROSPERO/display_record.php?RecordID=380958.

## Introduction

Sepsis refers to life-threatening organ dysfunction caused by a dysregulated host response to infection (1). Sepsis is a growing global health issue, with an estimated 49 million cases worldwide resulting in 11 million deaths. Despite a declining age-standardized incidence and mortality, sepsis remains a major cause of health loss worldwide. The incidence and mortality of sepsis vary substantially across regions, with the highest burden in sub-Saharan Africa, Oceania, south Asia, east Asia, and southeast Asia (2, 3). Data from high-income countries indicate that the number of sepsis-related deaths was 2.8 million per year (4). Therefore, there is an urgent need for a new treatment option or adjuvant treatments to improve the clinical outcome of sepsis.

Ascorbic acid, usually called vitamin C, is a crucial trace element in the human body. In terms of pathophysiology, vitamin C as an antioxidant, protects essential molecules in the body from damage caused by free radicals and reactive oxygen species (ROS) produced by active immune cells (5–8). Furthermore, vitamin C is involved in the synthesis of catecholamine hormones and amide peptide hormones which are the cores of cardiovascular response to severe infections (9, 10). Moreover, vitamin C improves microvascular reactivity and peripheral tissue perfusion in septic shock patients (11). According to the previously published studies, an IV dose of 2 to 3 gram/day seems necessary to reach a normal plasma concentration (50–70 μmol/L) (12–14). However, higher doses greater than 6 grams per day may lead to greater benefits, including reduced use of vasopressors, faster recovery and lower mortality (15).

However, in the latest published LOVIT study, the composite outcome of death or persistent organ dysfunction on trial day 28 was higher in patients administered vitamin C than in the control group [risk ratio (RR), 1.21; 95% confidence interval (CI), 1.04 to 1.40; *p* = 0.01]. To explore this controversial issue, we conducted a meta-analysis of randomized controlled trials to assess the effect of intravenous vitamin C as an adjuvant treatment option in patients with sepsis.

## Methods

This study was conducted according to the Preferred Reporting Items for Systematic Reviews and Meta-Analyses guidelines (PRISMA) and was registered in PROSPERO (registration number: CRD42022380958) (16, 17).

### Eligibility criteria

A systematic literature search was completed for all peer reviewed and published studies reporting the effects of intravenous vitamin C treatment compared to standard care or placebo treatment. The inclusion criteria were as follows: (1) Population: adult patients (≥18 years of age) with sepsis; (2) Intervention: Intravenous vitamin C as the treatment group; (3) Comparison: placebo or standard care; (4) Outcomes: a primary outcome of all-cause short-term mortality. In the present meta-analysis, we defined all-cause short-term mortality as in-hospital mortality or 28-day/30-day mortality. Secondary outcomes included the duration of vasopressor use, length of ICU stay and SOFA score (according to the end time of administration), C-reactive protein (CRP) levels; (5) Study type: randomized controlled trials were eligible for inclusion.

### Data extraction

Two authors (Xing Luo, Jianqiu Zhu) independently retrieved relevant studies. We extracted the characteristics (first author, year of publication, study design, study site, participants, number of participants, intervention, control methods and vitamin C dose) and predefined outcomes from the included studies.

### Assessment of risk of bias

Two investigators (Xing Luo, Jianqiu Zhu) independently assessed the risk of bias for each of the included studies using the Cochrane risk-of-bias tool (18). Any differences in opinion were resolved by a third adjudicator (Youfeng Zhu).

### Statistical analysis

Statistical analysis was performed using Review Manager version 5.4. We calculated the odds ratio (OR) with 95% confidential interval (CI) for categorical variables and the mean difference (MD) with 95% CI for continuous variables. Continuous variables, such as the duration of vasopressor use, length of ICU stay and SOFA score, which are expressed in the form of median and interquartile ranges, were assessed for skewness and normality of distribution, and then we converted these data into mean and standard deviation according to previously published studies (19, 20). Random-effects models were used to pool the data. Heterogeneity was assessed using the I^2^ statistic. I^2^ > 50% or *p* < 0.10 was considered to indicate substantial heterogeneity. Finally, sensitivity analysis was conducted to explore the effect of individual studies by consecutive exclusion of one study at a time.

### Subgroup analysis

According to the previously published studies, the dose and treatment duration might influence the effect of vitamin C. Hence, we conducted subgroup analyses according to the dose and duration of administration of vitamin C and region (developed countries versus developing countries).

## Results

**Figure 1:**
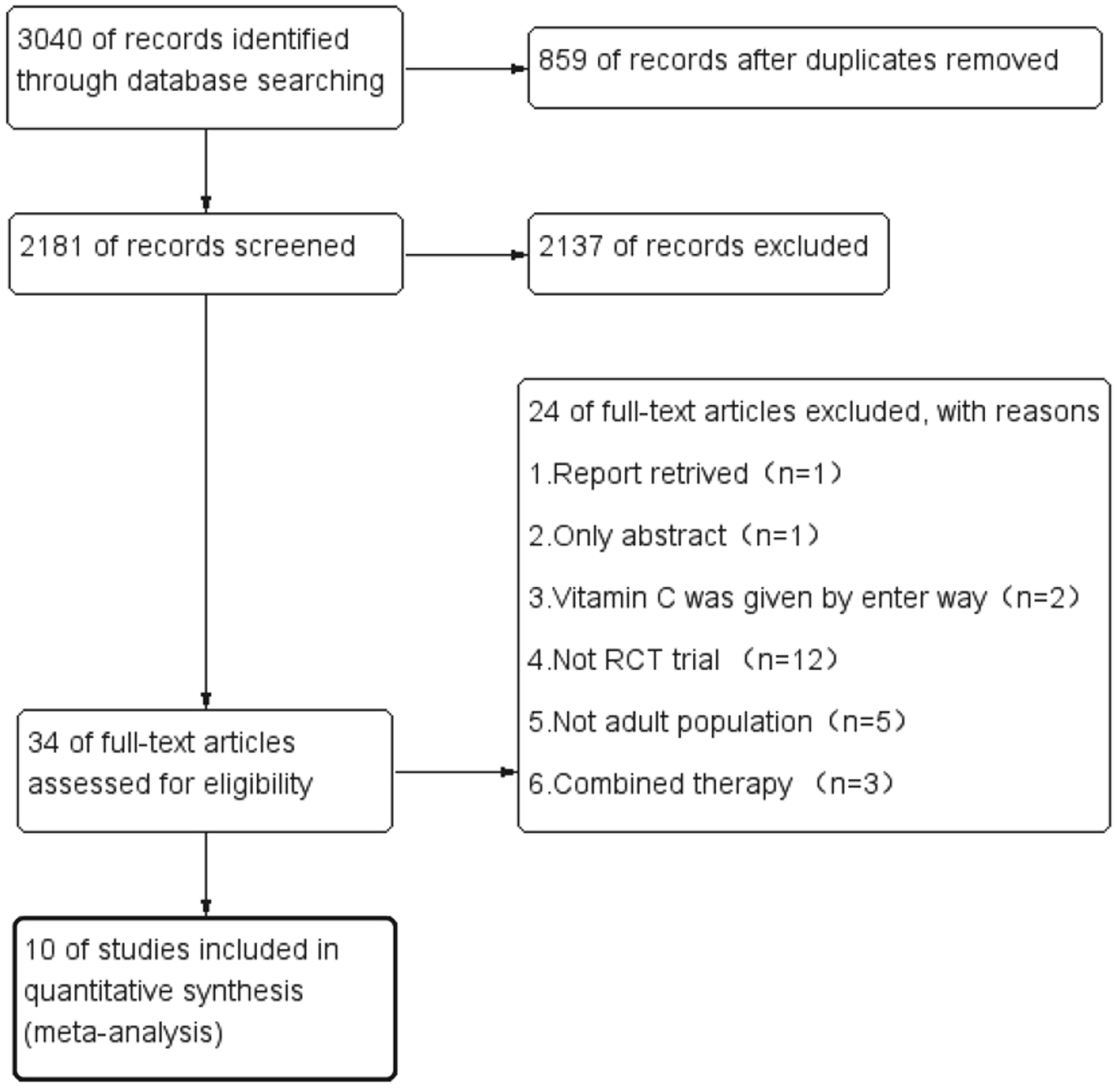
PRISMA flow diagram showing search and selection study selection strategies.

During the initial search, we found 3,040 articles. Among them, 859 were duplicated articles, and 2,137 additional studies were excluded by screening the abstracts. During the evaluation of the full text, 24 studies were further removed for various reasons (Figure 1). Finally, a total of ten randomized controlled trials were included in our study (21–30). The study selection flow diagram is visualized in Figure 1.

Table 1 presents the characteristics of the included studies. A total of 1,426 patients were included in the analysis, 713 of whom received intravenous (IV) vitamin C intervention during the study period, while 713 received placebo treatment. However, initiation time, dose of vitamin C, duration of the intervention and population type varied among the included trials. Two trials administered low-dose vitamin C (21, 26), and seven studies administered high-dose vitamin C (23–25, 27–30). One trial included a low-dose vitamin C group (less than or equal to 100 mg/kg/day) and a high-dose vitamin C group (greater than 100 mg/kg/day) (22). In eight trials, patients received IV vitamin C for less than 5 days (22–24, 26–30), and two trials administered vitamin C for 5 days or more (21, 25). There were six studies with patients from developed countries (21, 22, 24, 28–30) and four studies with patients from developing countries (23, 25–27).

**Table 1:** Characteristics of included studies.

Study	Study design	Study site	Sample size	Population	Interventions	Comparator	Outcomes
Lamontagne, 2022	Double-blind, randomized placebo-controlled trial	35 ICUs in Canada, France, and New Zealand.	Total: 863 (Intervention: 429; Control: 434)	Patients with sepsis requiring vasopressor	vitamin C (200 mg/kg/day) for 4 days;	5% dextrose or normal saline	composite of death or persistent organ dysfunction,SOFA score at 4-day
Rosengrave, 2022	Double-blind, randomized placebo-controlled trial	1 ceter in New Zealand	Total: 40 (Intervention: 20; Control: 20)	Adult patients with septic shock	vitamin C (100 mg/kg/day) for 4 days;	5% dextrose	Mortality (in-hospital, 30-day, 90-day), duration of vasopressor, ICU
Wacker, 2022	Double-blind, randomized placebo-controlled trial	8 ceters in US	Total: 124 (Intervention: 60; Control: 64)	Adult patients within 24 h of onset of septic shock	vitamin C (6,000 mg/day) for 4 days;	normal saline	28-day mortality, duration of vasopressor, ICU length of stay
Driny, 2022	Double-blind, randomized placebo-controlled trial	1 ceter in Egypt	Total: 40 (Intervention: 20; Control: 20)	Adult patients with sepsis requiring mechanical ventilation	vitamin C (6,000 mg/day) for 4 days;	normal saline	28-day mortality, duration of vasopressor, ICU length of stay
Gayathri Ranie, 2021	A randomized placebo-controlled trial	1 ceter in india	Total: 40 (Intervention: 20; Control: 20)	Patients with sepsis shock	vitamin C (6,000 mg/day) for 5 days;	5% dextrose	In-hospital mortality
Fowler, 2019	Double-blind, randomized placebo-controlled trial	7 ceters in US	Total: 167 (Intervention: 84; Control: 83)	Patients with sepsis and ARDS	vitamin C (50 mg/kg/day or 200 mg/kg/day) for 4 days;	5% dextrose	28-day mortality, improvement in SOFA score, SOFA score at 4-day
Zabet, 2016	Double-blind, randomized placebo-controlled trial	1 ceter in Iran	Total: 28 (Intervention: 14; Control: 14)	Adult surgical critically ill patients with diagnosis of septic shock	vitamin C (100 mg/kg/day) for 3 days;	5% dextrose	28-day mortality, duration of vasopressor, ICU length of stay
Fowler, 2014	Double-blind, randomized placebo-controlled trial	1 ceter in US	Total: 24 (Intervention: 16; Control: 8)	Adult patients with severe sepsis in the ICU	vitamin C (200 mg/kg/day) for 4 days;	5% dextrose	28-day mortality, improvement in SOFA score, SOFA score at 4-day
Mahmoodpoor, 2021	Double-blind, randomized placebo-controlled trial	1 ceter in Iran	Total: 80 (Intervention: 40; Control: 40)	Critically ill patients with severe pneumonia	vitamin C (60 mg/kg/day) for 4 days;	normal saline	In-hospital mortality, duration of vasopressor, SOFA score at 4-day, ICU length of stay
Ferrón-Celma, 2009	Double-blind, randomized placebo-controlled trial	1 ceter in Spain	Total: 20 (Intervention: 10; Control: 10)	Adult patients developed sepsis after abdominal surgery	vitamin C (450 mg/day) for 6 days;	5% dextrose	In-hospital mortality

### Quality assessment

**Figure 2:**
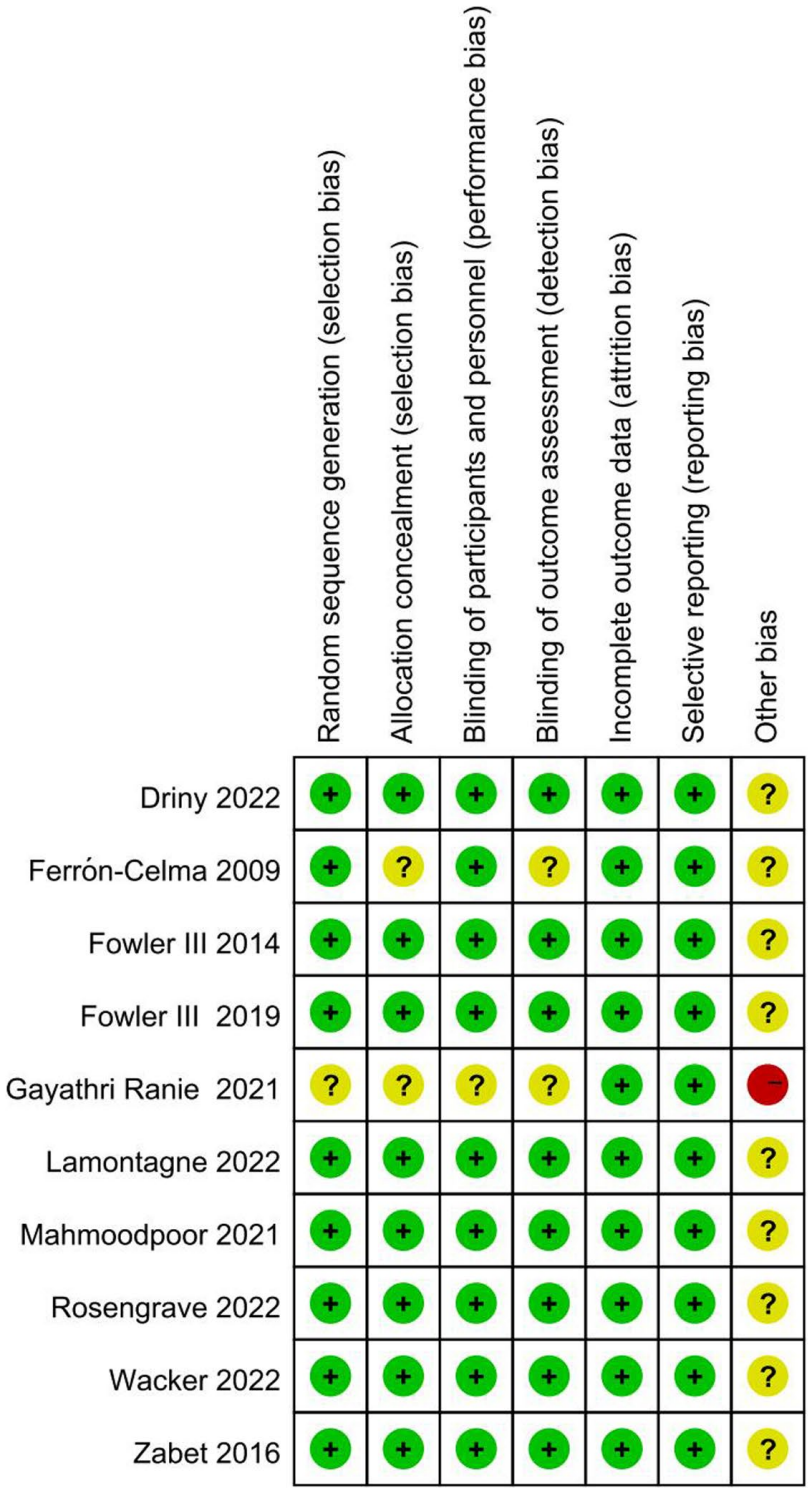
Risk of bias assessment.

The results of the risk of bias assessment (Figure 2) showed that one study was rated as having a high risk of bias. Gayathri Ranie et al. used an open-label design, which carried a risk of bias (25). Two trials (21, 25) did not provide the methods of random sequence generation or allocation concealment. Two trials did not report the blinding method or may have broken the blinding, which would either underestimate or overestimate the size of the effect (21, 25). Moreover, all ten trials were rated as having an unclear risk of other bias, as these studies did not account for disease heterogeneity in patients with sepsis and did not analyze septic patients with specific phenotypes based on whether they were associated with a high inflammatory state, reduced vascular reactivity, or vitamin C deficiency.

### Primary outcome

**Figure 3:**
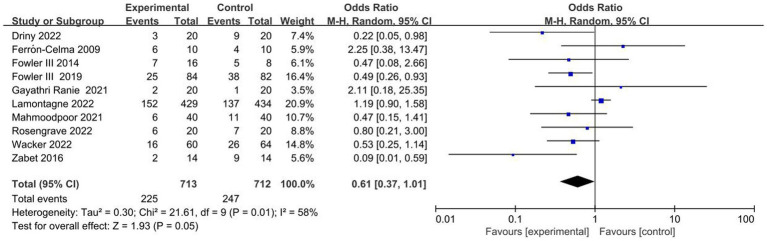
Meta-analysis of short-term mortality in patients receiving vitamin C therapy versus placebo.

The ten included trials reported short-term mortality with different definitions. Six trials (22–24, 27, 28, 30) reported 28-day mortality, three trials (21, 25, 26) reported in-hospital mortality, and one trial (29) reported multiple results; Overall, no significant difference in short-term mortality was observed between the study groups (OR, 0.61; 95% CI: 0.37–1.01; *p* = 0.05) (Figure 3).

### Secondary outcomes

**Figure 4:**
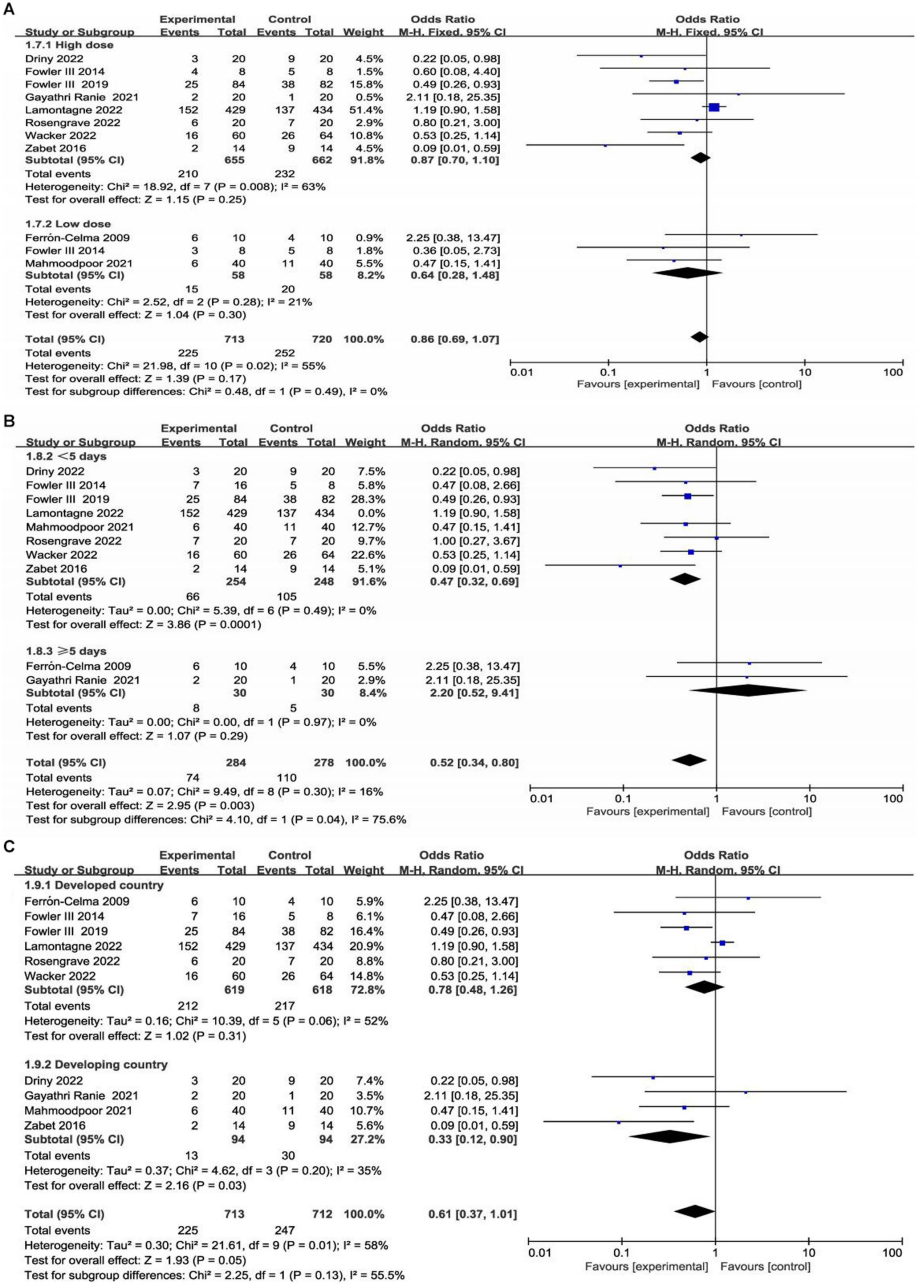
Comparison of vitamin C versus placebo with regards to duration of vasopressor use **(A)**, Length of ICU stay **(B)** and SOFA score **(C)**.

Five studies reported the duration of vasopressor use (23, 26, 27, 29, 30), the length of ICU stay (23, 26, 27, 29, 30) and the SOFA score (24, 26–29). Vitamin C treatment was associated with a reduction in the duration of vasopressor use (MD, −14.36; 95% CI, −26.11 to −2.61; *p* = 0.02, Figure 4A).

However, there was no significant difference in the length of ICU stay (MD, −1.24, 95% CI, −3.54 to 1.05, *p* = 0.29, Figure 4B) or SOFA score (MD, −0.85, 95% CI, −2.38 to 0.67, *p* = 0.27, Figure 4C) between the groups. Furthermore, there was high heterogeneity among the included studies.

**Figure 5:**
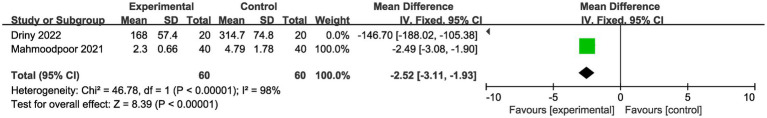
Comparison of CRP level between vitamin C group and placebo group. CRP: C-reactive protein.

Meanwhile, we performed the comparison of CRP levels between vitamin C group and placebo group (Figure 5). The result showed that there was a significant reduction for CRP levels in vitamin C group compared with control group (MD -2.52, 95%CI -3.11--1.93, *p* < 0.001, Figure 5). However, the doses used in the involved studies were different. The doses of vitamin C used in one study (27) was 6,000 mg/day, while it was 60 mg/kg/day in another study (26). Hence, further large scale studies are needed for investigating the optimal doses of vitamin C in sepsis patients.

### Subgroup analysis

We conducted subgroup analyses according to the dose and duration of use of vitamin C and region (developed countries versus developing countries). Detailed data are shown in the Supplementary materials. Two studies enrolled low-dose vitamin C (21, 26), and seven studies enrolled high-dose vitamin C (23–25, 27–30). One trial included a low-dose vitamin C group and a high-dose vitamin C group (22). In eight trials, patients received vitamin C for less than 5 days (22–24, 26–30), and two trials administered vitamin C for 5 days or more (21, 25). In addition, there were six studies with patients from developed countries (21, 22, 24, 28–30) and four studies with patients from developing countries (23, 25–29).

Interestingly, our subgroup analysis showed that vitamin C treatment led to a significant short-term mortality reduction in patients from developing countries (OR, 0.33; 95% CI, 0.12–0.90, *p* = 0.03; Supplementary Figure S3). Furthermore, subgroup analysis found that in developing countries, vitamin C was associated with a significant reduction in the duration of vasopressor use (MD, −24.37, 95% CI -33.72 to −15.02, *p* < 0.001; Supplementary Figure S4) and SOFA score (MD, −2.55, 95% CI -4.81 to −0.28, *p* = 0.03; Supplementary Figure S5).

## Discussion

In this meta-analysis, we included ten randomized controlled trials involving 1,426 patients to analyze the effect of intravenous vitamin C as an adjunctive option for treatment in sepsis patients. To our knowledge, this is the latest meta-analysis regarding this topic to date. Our study showed that vitamin C administration for sepsis patients was not associated with a significant reduction in short-term mortality, length of ICU stay or SOFA score. However, we observed that vitamin C could reduce the duration of vasopressor use, which could be explained by the mechanism of action of vitamin C.

For a long time, vitamin C was shown to play an important role in the control of free radicals and ROS (31–34). Sepsis can lead to the release of several ROS, which are capable of causing severe injury to lipids, proteins, and nucleic acids that, in turn, results in endothelial and mitochondrial dysfunction, cell death, and ultimately multiple organ dysfunction syndrome (MODS) (14, 20, 35). Vitamin C exerts its antioxidant effects by scavenging these ROS. In addition, vitamin C is an important antioxidant in the body, supporting the synthesis of vasopressin, cortisol and catecholamine in the form of coenzymes. Vitamin C also has several immunomodulatory and anti-inflammatory effects that deficient levels correlated with multiple organ dysfunction failure. This compound also enhances the synthesis of these enzymes and improves arteriolar sensitivity to vasopressors by inhibiting endothelial expression of inducible nitric oxide synthase (iNOS) (9, 11, 33, 34, 36, 37). This might be the reason why vitamin C reduced the duration of vasopressor use according to the analyses in our study.

Vitamin C deficiency might be contributing to the complications of sepsis. Two included studies reported the baseline vitamin C levels (22, 25). In the one study (25), the normal range of Vitamin C was 4.4 to 14.4 ng/mL. And the baseline Vitamin C level was significantly lower in sepsis patients (5.14 ± 4.19 ng/mL) than that in healthy controls (14.64 ± 5.51 ng/mL). And according to the other study (22), the plasma vitamin C level of septic patients was 17.9 ± 2.4 μM, while it was 50–70 μM in normal populations. In an observational study, it was estimated that nearly 40% sepsis patients were vitamin C deficient (14).

Subgroup analysis showed that sepsis patients in developing countries could benefit more from vitamin C supplementation and could serve as a way identify specific sepsis patients. To our knowledge, this is the first study that found the outcomes of sepsis patients in developing countries were significantly improved with vitamin C administration. This might be because patients in developing countries usually receive insufficient vitamin C. Previous studies have shown that the vitamin C level in people of developing countries is generally lower than that in developed countries, and there is a high prevalence of vitamin C deficiency, particularly in low- and middle-income countries, so sepsis patients in developing countries may need more vitamin C supplements (38, 39). As few included RCTs reported the vitamin C levels, a further meta-analysis with regards to the baseline vitamin C level could not be performed.

Previous study showed that vitamin C played a various roles in the immune system, including maintaining mucosal barrier integrity and leukocyte function (40–42). Certain cell types accumulate high concentrations of vitamin C via the sodium-dependent vitamin C transporter (SVCT). Although physiological plasma concentrations of vitamin C are 50–70 umol/L, concentrations in some organs are more higher (1 mmol/L in the liver and lung, 2–10 mmol/L in the brain and 4–10 mmol/L in the adrenal glands) (40). Leukocytes actively accumulate vitamin C via SVCT, resulting in a concentration over 1 mM and increasing the concentration to 10 mM after an oxidative burst. This protects immune cells from self-induced oxidative bursts caused by killing pathogens (40). However, the appropriate doses of vitamin C for immune system are still un-conclusive. As few included RCTs reported the vitamin C levels within immune system, a further meta-analysis with regards to the optimal doses for the immune system could not be performed.

The recent LOVIT study showed that the use of vitamin C in the treatment of sepsis was harmful to the composite outcome (death or persistent organ dysfunction) on trial day 28 (risk ratio, 1.21; 95% confidence interval [CI], 1.04 to 1.40; *p* = 0.01) (24). This result needs to be treated with great caution. Most importantly, a composite outcome (death or persistent organ dysfunction) rather than simple mortality or other survival indicator was used as the primary outcome in the LOVIT study, which was different from previous studies. For sepsis patients requiring vasoactive drug treatment, prognostic indicators such as 28-day mortality or MODS are affected by many factors, which may affect the evaluation of the effectiveness of vitamin C.

However, this meta-analysis also has several limitations. First, since sepsis is a clinically common syndrome with high heterogeneity, the patients included in the present study represent a heterogeneous population. For example, some were surgical patients who had undergone a major operation, some had severe pneumonia or respiratory failure, and some were patients with the latest form of COVID-19 (21–30). Second, vitamin C deficiency could be contributing to the complications of sepsis. As few included RCTs reported the vitamin C levels, a further meta-analysis with regards to the baseline vitamin C level could not be performed. Third, most of the outcome analyses were based on a small population sample size, and single-center trials showed larger treatment effects than multicenter trials. These may generate bias. Finally, the safety of vitamin C was not comprehensively investigated in this study.

## Conclusion

Our study showed that vitamin C administration for sepsis patients was not associated with a significant reduction in short-term mortality, length of ICU stay or SOFA score. However, we observed that vitamin C can reduce the duration of vasopressor use. Furthermore, sepsis patients in developing countries may benefit more than those in developed countries. Our results need to be further confirmed by large-scale, randomized studies.

## Data availability statement

The original contributions presented in the study are included in the article/Supplementary material, further inquiries can be directed to the corresponding authors.

## Ethics statement

Ethical approval was not required for the study involving humans in accordance with the local legislation and institutional requirements. Written informed consent to participate in this study was not required from the participants or the participants’ legal guardians/next of kin in accordance with the national legislation and the institutional requirements.

## Author contributions

XL, YZ, RZ, JZ, HK, YS, XG, and BN contributed to the study conception and design. YZ, XL, JZ, RZ, YS, XG, and BN performed the research. YZ, XL, JZ, and RZ collected and analyzed the data. YZ, XL, JZ, RZ, YS, XG, and BN wrote the manuscript. All authors contributed to the article and approved the submitted version.

## Funding

This study was supported by the Health Science and Technology Project of Guangzhou Municipal Health Commission (20201A011022) and Research-oriented Hospital Program of Guangzhou (RHPG05) and Guangdong Natural Science Foundation (2020A1515010816). The funding body was not involved in the design of the study and collection, analysis, and interpretation of data and in writing the manuscript.

## Conflict of interest

The authors declare that the research was conducted in the absence of any commercial or financial relationships that could be construed as a potential conflict of interest.

## Publisher’s note

All claims expressed in this article are solely those of the authors and do not necessarily represent those of their affiliated organizations, or those of the publisher, the editors and the reviewers. Any product that may be evaluated in this article, or claim that may be made by its manufacturer, is not guaranteed or endorsed by the publisher.
