# Supplementary material for: The effect of vitamin C in adults with sepsis: a meta-analysis of randomized controlled trials
Source: Front Med (Lausanne). 2023 Aug 31;10:1244484. doi: 10.3389/fmed.2023.1244484 (PMC10502229; doi:10.3389/fmed.2023.1244484)
Supplement: Supplementary file 1 [file Data_Sheet_1.DOCX]

**Supplementary files**

subgroup analyses and Searching strategies


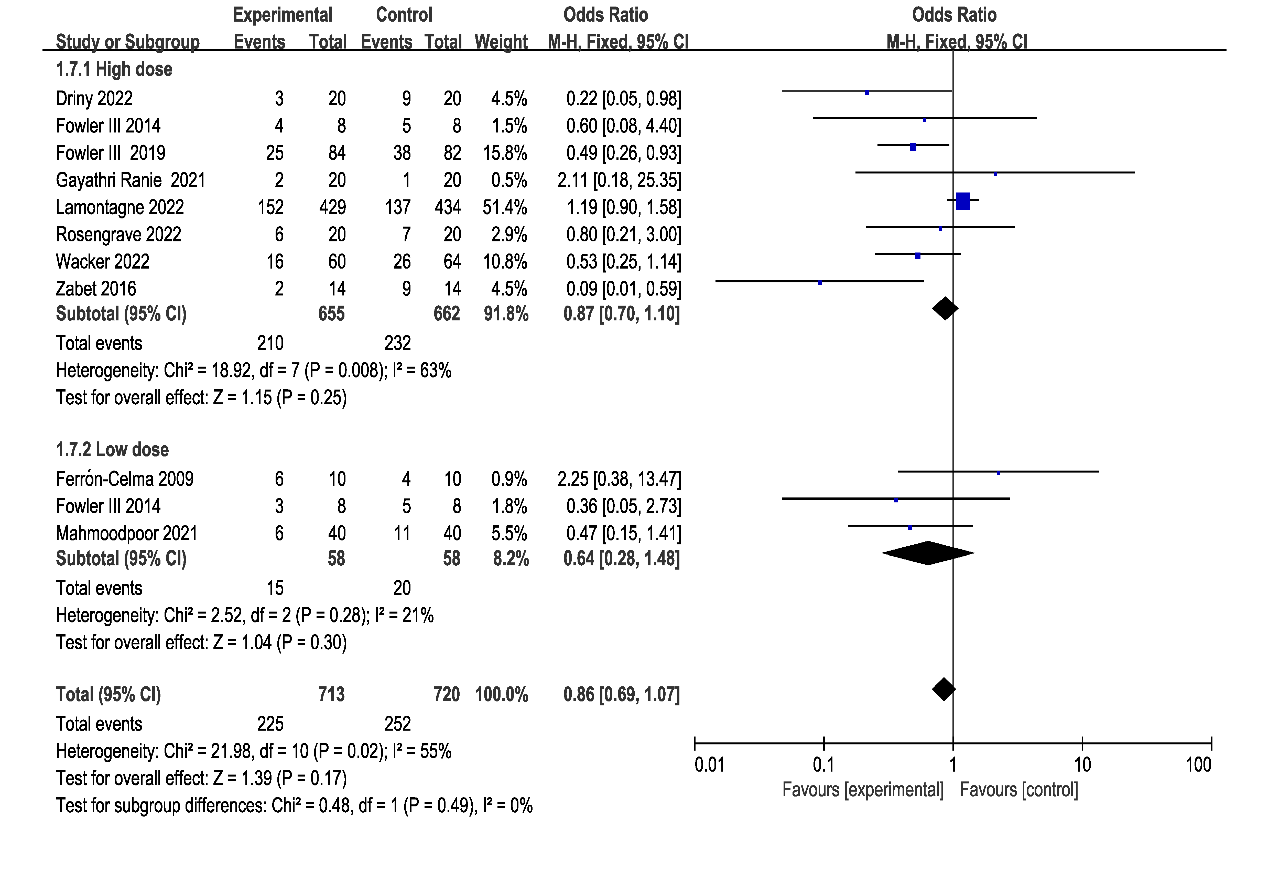


Figure S1. Subgroup analysis for short-term mortality, low dose versus high dose


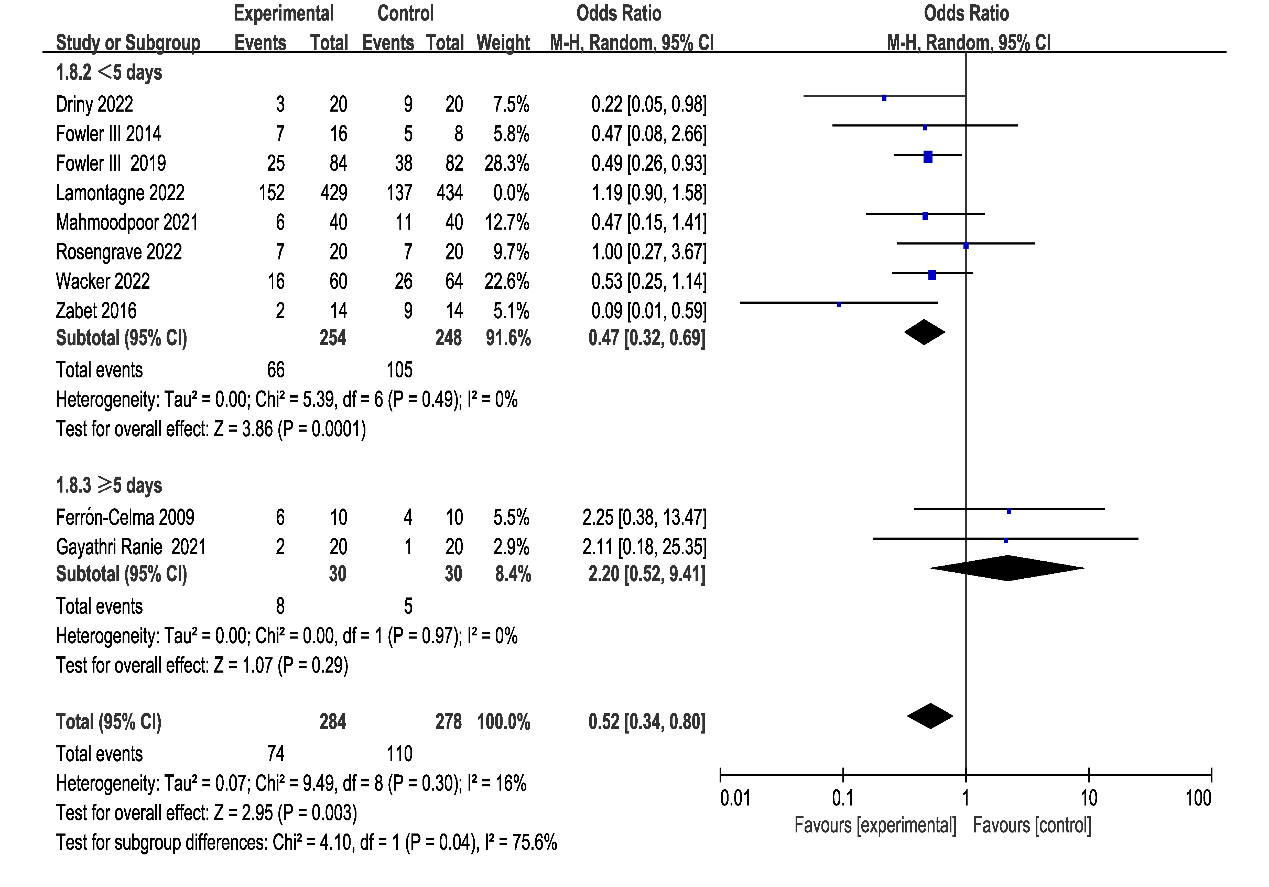


Figure S2.Subgroup analysis for short-term mortality, <5 days versus ≥5 days


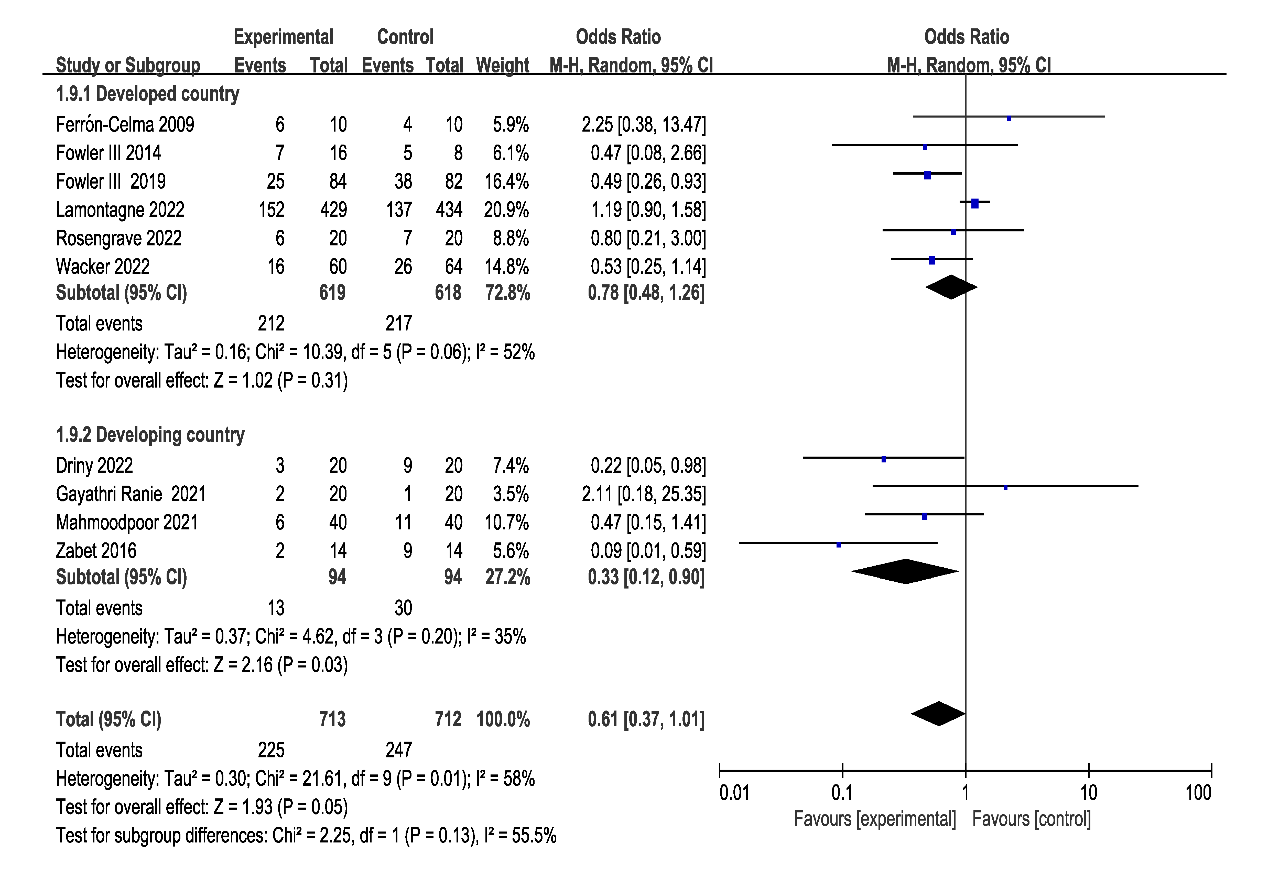


Figure S3. Subgroup analysis for short-term mortality, developed countries versus developing countries


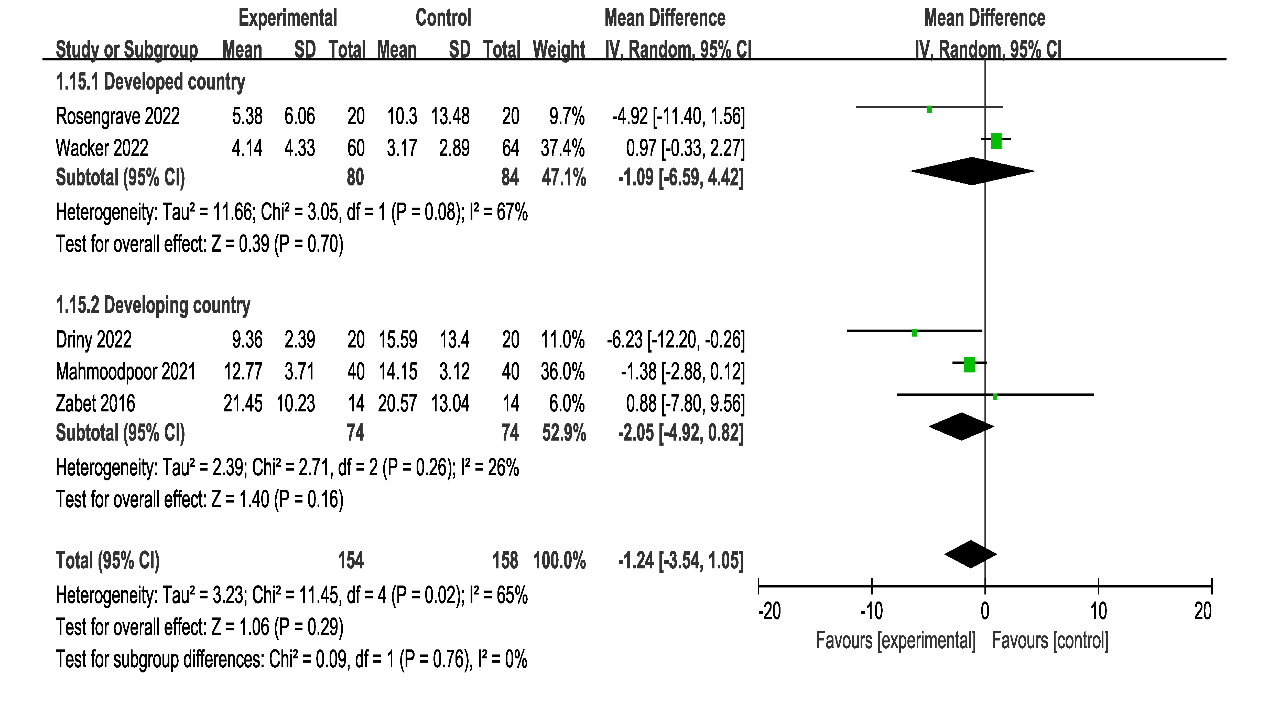


Figure S4. Subgroup analysis for length of ICU stay, developed countries versus developing countries


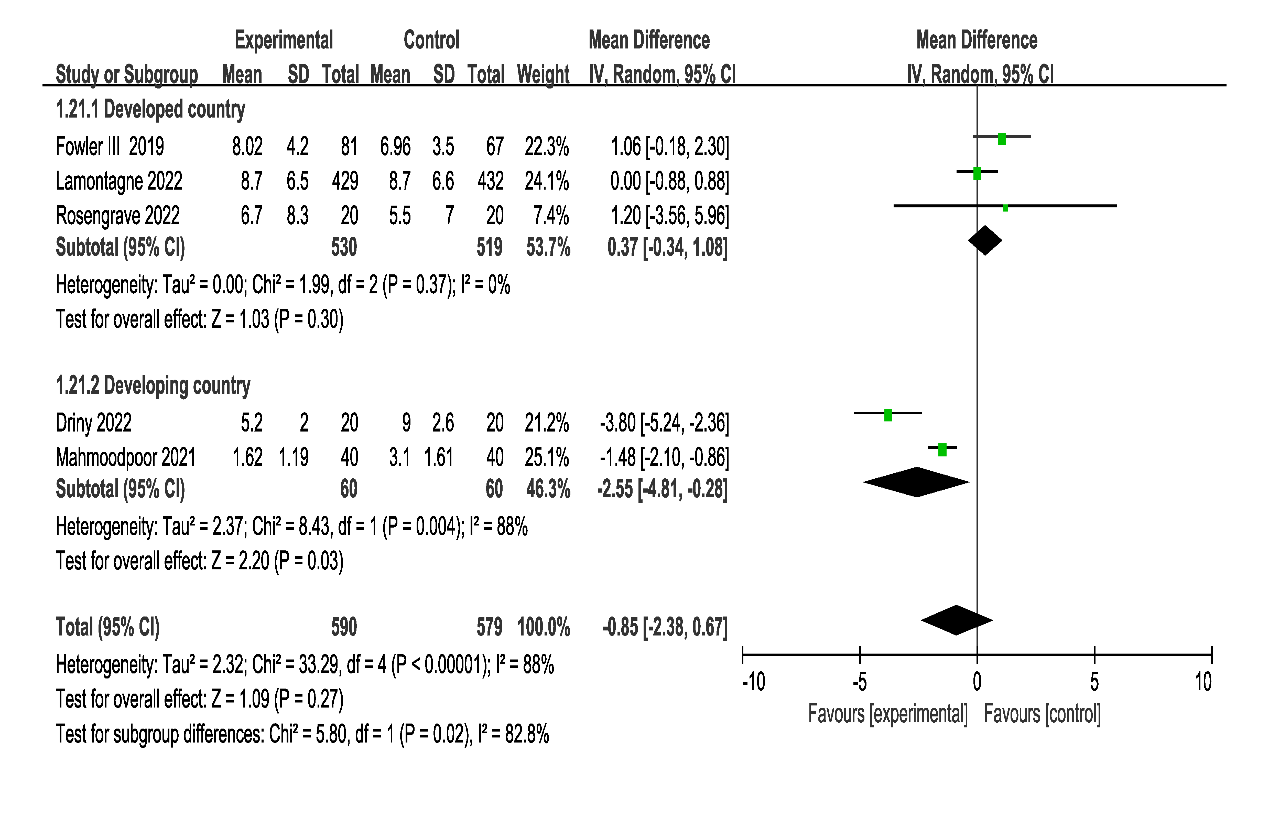


Figure S5. Subgroup analysis for SOFA score,developed countries versus developing countries


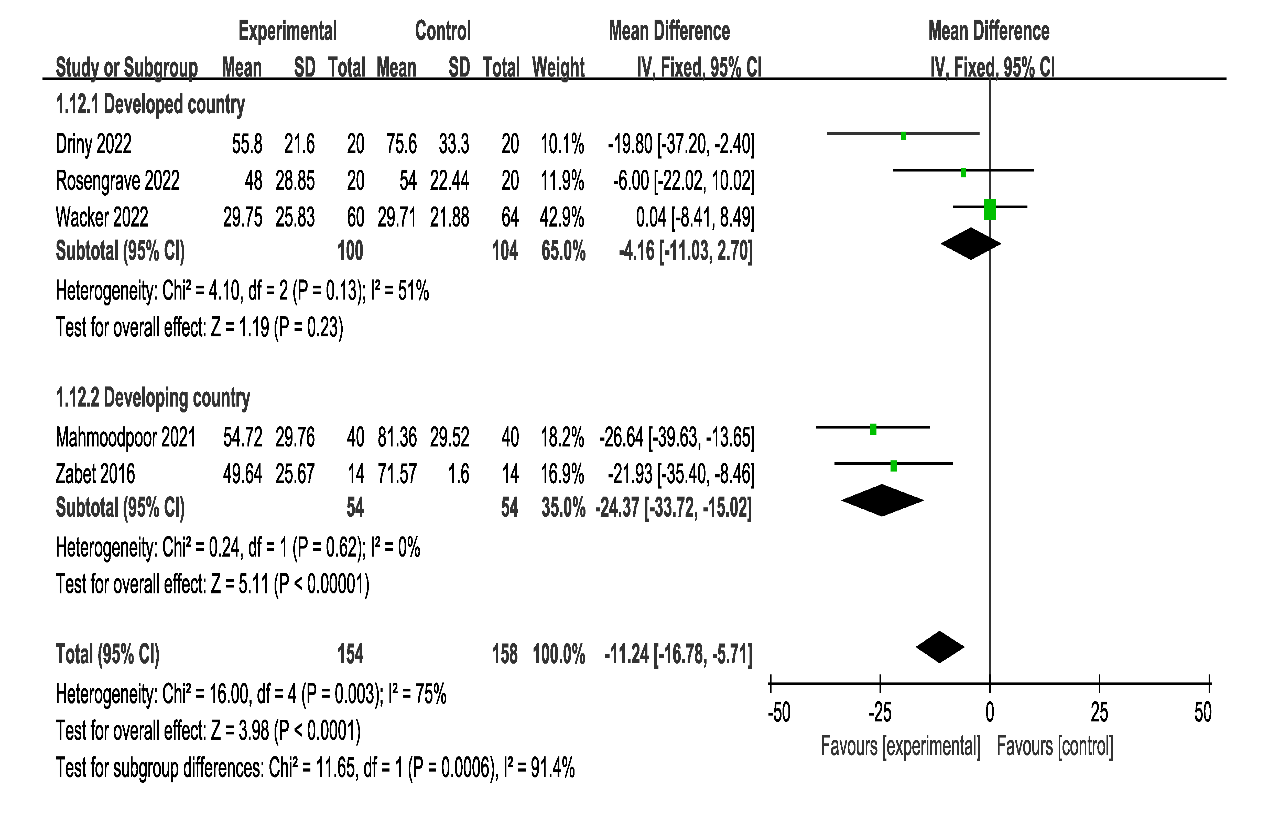


Figure S6. Subgroup analysis for duration of vasopressor use, developed countries versus developing countries

**Search strategy**

**Pubmed (result 520)**

(("Ascorbic Acid"[Mesh]) OR (((((((((((((((((((Ascorbic Acid[Title/Abstract]) OR (Acid, Ascorbic[Title/Abstract])) OR (L-Ascorbic Acid[Title/Abstract])) OR (Acid, L-Ascorbic[Title/Abstract])) OR (L Ascorbic Acid[Title/Abstract])) OR (Vitamin C[Title/Abstract])) OR (Hybrin[Title/Abstract])) OR (Magnorbin[Title/Abstract])) OR (Sodium Ascorbate[Title/Abstract])) OR (Ascorbate, Sodium[Title/Abstract])) OR (Ascorbic Acid, Monosodium Salt[Title/Abstract])) OR (Ferrous Ascorbate[Title/Abstract])) OR (Ascorbate, Ferrous[Title/Abstract])) OR (Magnesium Ascorbate[Title/Abstract])) OR (Ascorbate, Magnesium[Title/Abstract])) OR (Magnesium di-L-Ascorbate[Title/Abstract])) OR (Magnesium di L Ascorbate[Title/Abstract])) OR (di-L-Ascorbate, Magnesium[Title/Abstract])) OR (Magnesium Ascorbicum[Title/Abstract]))) AND (((((((((((((((((("Sepsis"[Mesh]) OR (Bloodstream Infection[Title/Abstract])) OR (Bloodstream Infections[Title/Abstract])) OR (Infection, Bloodstream[Title/Abstract])) OR (Pyemia[Title/Abstract])) OR (Pyemias[Title/Abstract])) OR (Pyohemia[Title/Abstract])) OR (Pyohemias[Title/Abstract])) OR (Pyaemia[Title/Abstract])) OR (Pyaemias[Title/Abstract])) OR (Septicemia[Title/Abstract])) OR (Septicemias[Title/Abstract])) OR (Blood Poisoning[Title/Abstract])) OR (Blood Poisonings[Title/Abstract])) OR (Poisonings, Blood[Title/Abstract])) OR (Poisoning, Blood[Title/Abstract])) OR (Severe Sepsis[Title/Abstract])) OR (Sepsis, Severe[Title/Abstract]))

**Cochrane (result 241)**

（Ascorbic Acid OR Acid, Ascorbic OR L-Ascorbic Acid OR Acid, L-Ascorbic OR L Ascorbic Acid OR Vitamin C OR Hybrin OR Magnorbin OR Sodium Ascorbate OR Ascorbate, Sodium OR Ascorbic Acid, Monosodium Salt OR Ferrous Ascorbate OR Ascorbate, Ferrous OR Magnesium Ascorbate OR Ascorbate, Magnesium OR Magnesium di-L-Ascorbate OR Magnesium di L Ascorbate OR di-L-Ascorbate, Magnesium OR Magnesium Ascorbicum）：ab,ti,kw

(Sepsis OR Bloodstream Infection OR Bloodstream Infections OR Infection, Bloodstream OR Pyemia OR Pyemias OR Pyohemia OR Pyohemias OR Pyaemia OR Pyaemias OR Septicemia OR Septicemias OR Blood Poisoning OR Blood Poisonings OR Poisonings, Blood OR Poisoning, Blood OR Severe Sepsis OR Sepsis, Severe ):ab,ti,kw

MeSH descriptor: [Ascorbic Acid] explode all trees

MeSH descriptor: [Sepsis] explode all trees

**Embase (result 1311)**

‘Bloodstream Infection’:ab,ti OR ‘Bloodstream Infections’:ab,ti OR ‘Infection, Bloodstream’:ab,ti OR ‘Infection, Bloodstream’:ab,ti OR ‘Pyemia’:ab,ti OR ‘Pyemias’:ab,ti OR ‘Pyohemia’:ab,ti OR ‘Pyohemias’:ab,ti OR ‘Pyaemia’:ab,ti OR ‘Pyaemias’:ab,ti OR ‘Septicemia’:ab,ti OR ‘Septicemias’:ab,ti OR ‘Blood Poisoning’:ab,ti OR ‘Blood Poisonings’:ab,ti OR ‘Poisonings, Blood’:ab,ti OR ‘Poisoning, Blood’:ab,ti OR ‘Severe Sepsis’:ab,ti OR ‘Sepsis, Severe’:ab,ti

‘Acid, Ascorbic’:ab,ti OR ‘L-Ascorbic Acid’:ab,ti OR ‘Acid, L-Ascorbic’:ab,ti OR ‘L Ascorbic Acid’:ab,ti OR ‘Vitamin C’:ab,ti OR ‘Vitamin C’:ab,ti OR ‘Vitamin C’:ab,ti OR ‘Sodium Ascorbate’:ab,ti OR ‘Ascorbate, Sodium’:ab,ti OR ‘Ascorbic Acid, Monosodium Salt’:ab,ti OR ‘Ferrous Ascorbate’:ab,ti OR ‘Ascorbate, Ferrous’:ab,ti OR ‘Magnesium Ascorbate’:ab,ti OR ‘Ascorbate, Magnesium’:ab,ti OR ‘Magnesium di-L-Ascorbate’:ab,ti OR ‘Magnesium di L Ascorbate’:ab,ti OR ‘di-L-Ascorbate, Magnesium’:ab,ti OR ‘Magnesium Ascorbicum’:ab,ti

**Web of Science (result 968)**

TS=（Ascorbic Acid OR Acid, Ascorbic OR L-Ascorbic Acid OR Acid, L-Ascorbic OR L Ascorbic Acid OR Vitamin C OR Hybrin OR Magnorbin OR Sodium Ascorbate OR Ascorbate, Sodium OR Ascorbic Acid, Monosodium Salt OR Ferrous Ascorbate OR Ascorbate, Ferrous OR Magnesium Ascorbate OR Ascorbate, Magnesium OR Magnesium di-L-Ascorbate OR Magnesium di L Ascorbate OR di-L-Ascorbate, Magnesium OR Magnesium Ascorbicum）

TS=(Sepsis OR Bloodstream Infection OR Bloodstream Infections OR Infection, Bloodstream OR Pyemia OR Pyemias OR Pyohemia OR Pyohemias OR Pyaemia OR Pyaemias OR Septicemia OR Septicemias OR Blood Poisoning OR Blood Poisonings OR Poisonings, Blood OR Poisoning, Blood OR Severe Sepsis OR Sepsis, Severe )
